# Angiotensin-(1-7) improves oxygenation, while reducing cellular infiltrate and fibrosis in experimental Acute Respiratory Distress Syndrome

**DOI:** 10.1186/s40635-015-0044-3

**Published:** 2015-02-27

**Authors:** Vanessa Zambelli, Giacomo Bellani, Roberto Borsa, Federico Pozzi, Alice Grassi, Margherita Scanziani, Vittoria Castiglioni, Serge Masson, Alessandra Decio, John G Laffey, Roberto Latini, Antonio Pesenti

**Affiliations:** Department of Health Sciences, University of Milano-Bicocca, Monza, Italy; Department of Emergency, San Gerardo Hospital, Monza, Italy; Mouse & Animal Pathology Lab, Fondazione Filarete, Milan, Italy; Department of Cardiovascular Research, IRCCS - Istituto di Ricerche Farmacologiche Mario Negri, Milan, Italy; Department of Oncology, IRCCS - Istituto di Ricerche Farmacologiche Mario Negri, Milan, Italy; Keenan Research Centre for Biomedical Science, St. Michael’s Hospital, Toronto, ON Canada; Departments of Anesthesia and Physiology, University of Toronto, Toronto, ON Canada

**Keywords:** ARDS, Renin-angiotensin system, Angiotensin-(1-7)

## Abstract

**Background:**

The renin-angiotensin system (RAS) plays a role in the pathogenesis of ARDS, Angiotensin II (Ang-II) contributing to the pathogenesis of inflammation and fibrogenesis. Angiotensin-(1-7) (Ang-(1-7)) may antagonize the effects of Ang-II. This study was aimed at evaluating the potential for Ang-(1-7) to reduce injury, inflammation and fibrosis in an experimental model of ARDS in the acute and late phases.

**Methods:**

Male Sprague Dawley rats underwent an instillation of 0.1 M hydrochloric acid (HCl, 2.5 ml/kg) into the right bronchus. In an acute ARDS study, acid-injured rats were subjected to high stretch mechanical ventilation (18 ml/kg) for 5 h and randomized to receive an intravenous infusion of either vehicle (saline), Ang-(1-7) at low dose(0.27 μg/kg/h) (ALD), or high dose (60 μg/kg/h) (AHD) starting simultaneously with injury or 2 h afterwards. Arterial blood gas analysis and bronchoalveolar lavage (BAL) were performed to assess the injury. For the late ARDS study, after HCl instillation rats were randomized to either vehicle or high dose Ang-(1-7) (300 μg/kg/day) infused by mini osmotic pumps for two weeks, and lung hydroxyproline content measured.

**Results:**

In the acute ARDS study, Ang-(1-7) led to a significant improvement in oxygenation (PaO_2_/FiO_2_ : vehicle 359 ± 86; ALD 436 ± 72; AHD 44 442 ± 56; ANOVA *p* = 0.007) and reduced white blood cells counts (vehicle 4,519 ± 2,234; ALD 2,496 ± 621; AHD 2,744 ± 119/mm^3^; ANOVA *p* = 0.004). Only treatment with high dose Ang-(1-7) reduced inflammatory cell numbers in BAL (vehicle 127 ± 34; AHD 96 ± 34/ μl; *p* = 0.033). Interestingly also delayed administration of Ang-(1-7) was effective in reducing injury. In later ARDS, Ang-(1-7) decreased hydroxyproline content (649 ± 202 and 1,117 ± 297 μg/lung; *p* < 0.05).

**Conclusions:**

Angiotensin-(1-7), decreased the severity of acute lung injury and inflammation induced by combined acid aspiration and high stretch ventilation. Furthermore, continuous infusion of Ang-(1-7) reduced lung fibrosis 2 weeks following acid aspiration injury. These results call for further research on Ang-(1-7) as possible therapy for ARDS.

## Background

To date, no therapies are available to modify the clinical course and improve the outcome of Acute Respiratory Distress Syndrome (ARDS) [1]. The only supportive strategies are mechanical ventilation and conservative management of intravenous fluids, but prolonged ventilation could exacerbate a pre-existing lung injury and lead to ventilator-induced lung injury (VILI) [2].

The pathogenesis of ARDS can be influenced by the activation of the renin-angiotensin system (RAS), a well-known hormonal system involved in the regulation of blood pressure homeostasis and fluid and salt balance. The main effector of this system is angiotensin II (Ang-II), which derives from the proteolytic cleavage of angiotensin I (Ang-I) by angiotensin converting enzyme (ACE). Ang-II, in addition to its hemodynamic functions, is involved in inflammatory and fibrogenic processes [3-5]. Indeed the activation of Ang-II and its receptor angiotensin II receptor type 1 (AT_1_R) plays a role in the pathophysiology of several diseases, including atherosclerosis, myocardial infarction, stroke, diabetes, nephrosclerosis, and tumorigenesis [5]. An association has been demonstrated between ACE polymorphism and the susceptibility, progression and outcome in ARDS [6-9].

In parallel with ACE/Ang-II/AT_1_R axis, renin-angiotensin system has a counter-regulatory axis, which is composed by angiotensin-(1-7) (Ang-(1-7)), its receptor Mas, and the main rate limiting step ACE2 [10]. Ang-(1-7) is a biologically active heptapeptide released from the cleavage of Ang-II by ACE homolog ACE2 or from Ang-I by other peptidases [11-13]. It exerts its action via the Mas receptor, which is a non-AT_1_/AT_2_ G protein-coupled receptor. Ang-(1-7) mediates some ACE inhibitor-related effects: it opposes Ang-II actions, especially vasoconstriction [14], proliferation [15], and inflammation [16]. In a model of antigen-induced arthritis [17], Ang-(1-7) modulated rolling and adhesion of leukocytes to endothelium with improvement of joint hypernociception. Ang-(1-7) can inhibit leukocyte pro-inflammatory functions by activating Mas receptor on their surface [5]. In the context of ARDS, Imai et al. showed the crucial involvement of ACE2 in the pathogenesis of ARDS in three different experimental models [18]. In particular, ACE2 knockout mice showed greater lung elastance than control mice, worst oxygenation, and increased inflammation in experimental models of acute lung injury induced by acid aspiration or endotoxin (LPS) or caecal ligation and puncture (CLP) [18]. Moreover the administration of recombinant human ACE2 in wild-type mice attenuated acute lung injury, demonstrating the protective effects of ACE2 [18]. Other studies demonstrated that Ang-(1-7) acts also on lung remodeling and fibrosis development, Ang-(1-7) can enhance the apoptosis of fibrocytes, and reduces the expression of transforming growth factor β (TGF-β) and collagen deposition [16,19-22]. In a bleomycin-induced lung fibrosis model, the intratracheal administration of Ang-(1-7) through lentivirus led to a significant reduction in pulmonary fibrosis [23]. The potential for Ang-(1-7) to attenuate ARDS severity and lung fibrosis in preclinical ARDS models is not known.

Even if the beneficial effects of Ang-(1-7) in different models of ARDS have already been shown [24], we aimed to further exploit the therapeutic potential of Ang-(1-7) for ARDS, also administrating it with a delayed timing in the acute phase and for a prolonged period of 2 weeks to evaluate the impact of fibrosis. To the best of our knowledge, these aspects have not been addressed in previous studies. We hypothesized that, in early ARDS, Ang-(1-7) would reduce the severity of lung injury induced following the combined ‘insults’ of pulmonary acid aspiration followed by high stretch mechanical ventilation.

Prolonged ARDS requiring ongoing mechanical ventilation can induce lung fibrosis, which is a significant contributor to poor outcome in ARDS [25]. Given this, we further wished to determine the potential for Ang-(1-7) to attenuate the development of fibrosis in an experimental model of late ARDS induced by unilateral acid instillation [26]. We hypothesized that continuous Ang-(1-7) treatment could reduce the collagen deposition and improve long-term outcome.

## Methods

### Animals

Male Sprague Dawley rats (200 to 250 g) were obtained from Harlan Laboratories (Udine, Italy) and maintained under standard laboratory condition in Bicocca University in Monza (Italy). Procedures involving animals and their care were conducted in conformity with the institutional guidelines complying with national (D.L. n. 116, G.U., suppl. 40, 18 Febbraio 1992, Circolare n. 8, G.U., 14 Luglio 1994) and international laws and policies (EEC Council Directive 86/609, OJ L 358, 1, 12 December 1987; US National Research Council’s Guide for the Care and Use of Laboratory Animals, 2011). The study was approved by the ethical committee of our institution.

### Acute ARDS study: two-hit model

Animals were anesthetized with Ketamine 100 mg/kg and Xylazine 4 mg/kg (Ketavet 100, Intervet Productions, Aprilia, Latina, Italy; Rompun 2%, Bayer, Milano, Italy), orotracheally intubated and subjected to high stretch ventilation (Inspira ASV, Harvard Apparatus, Holliston, MA, USA) with the following parameters: tidal volume 18 ml/kg; respiratory rate 35/min; PEEP 2 to 2.5 cmH_2_O; inspiration to expiration ratio (I/E) 35%; fraction of inspired oxygen (FiO_2_) 0.5. At the beginning, after 3 h of treatment and at the end of the 5-h experiment, a recruitment maneuver (to 30 cmH_2_O for 10 s) was performed. Injury was induced by instillation of 0.1 M hydrochloric acid (HCl) (2.5 ml/kg) into the right bronchus through a PE10 catheter. Acid-induced damage in the right lung was confirmed by micro-computed tomography (see the ‘Assessment of injury’ section). When we identified that HCl instillation involved the contralateral lung, the animals were euthanized and excluded from analysis (approximately 2%). Anesthesia and paralysis were maintained throughout the experiment by infusion in the right carotid of Propofol 13 mg/kg/h and Ketamine 5 mg/kg/h and in the right jugular of Rocuronium bromide (Rocuronio, Fresenius Kabi Italia, Isola della Scala, Verona, Italy) 1.5 mg/kg/h and Ringer acetate 1.8 ml/h. A group of rats (*n* = 5), that received an instillation of 2.5 ml/kg of sterile saline (NaCl 0.9%) and underwent prolonged mechanical ventilation with same ventilation settings, was used as control group (CTRL). A group of healthy rats (*n* = 6) did not undergo any of the surgical interventions and was sacrificed (Healthy rats).

#### Series 1 - early therapy with Ang-(1-7)

Immediately after acid instillation, rats were randomized to receive intravenous treatment (left jugular vein) depending on the randomly assigned experimental group (infusion rate 200 μl/kg/h):

- Vehicle: rats treated with vehicle (NaCl 0.9%)
- Ang-(1-7) low dose (ALD): rats treated with Ang-(1-7) at a dosage of 0.27 μg/kg/h
- Ang-(1-7) high dose (AHD): rats treated with Ang-(1-7) at a dosage of 60 μg/kg/h

#### Series 2 - rescue therapy with Ang-(1-7)

In this series, rats underwent high stretch ventilation and acid instillation as described above. Two hours after HCl instillation, animals were randomized to ‘rescue’ treatment with as follows:

- Vehicle: rats treated with vehicle (NaCl 0.9%)
- Ang-(1-7) high dose: rats treated with Ang-(1-7) at a dosage of 100 μg/kg/h

In this series the total dose of Ang-(1-7) was the same as the AHD group in series 1.

### Hemodynamic monitoring

In both series, invasive arterial, central venous, and airway pressures were monitored using pressure transducers, which were interfaced to a PowerLab (AD Instruments, Colorado Springs, CO, USA) signal transduction unit.

### Assessment of injury (after 5 h of treatment in series 1 and 2)

Blood withdrawal: blood samples were collected at different time points: immediately before the treatment (start), after 3 h and at the end of the 5-h experiment (end), and they were collected from the right carotid artery to perform peripheral leukocyte (WBCs) counts.

Arterial blood gas analysis: at the same time points, gas exchange was assessed by measuring arterial pressure of oxygen (PaO_2_), arterial pressure of carbon dioxide (PaCO_2_), pH, and base excess (BE) with i-STAT portable analyzer (Oxford Instruments S.M., Burke and Burke, Menfis Biomedica, Milan, Italy).

Computed tomography (CT) scan: animals underwent two CT scans (Skyscan 1176, Bruker, Brussels, Belgium). The first scan was performed immediately after the acid instillation in order to verify the extent of injury and the selective instillation of acid into the right lung, with these parameters: exposure 65 ms, voltage 80 kV, current 300 μA, and resolution 35 μm (scanning duration about 5 min). At the end of the experiment, the second scan was performed in order to measure the amount of hypoaerated areas of parenchyma in the left and right lung separately. This scanning (taking about 20 min) was gated on respiratory cycles.

Respiratory system compliance: respiratory system static compliance was measured every hour during mechanical ventilation by end-inspiratory occlusions and calculated according to standard formulas.

Broncho-alveolar lavage (BAL): rats were euthanized (via exsanguination), and BAL was performed in right and left lung separately. Total protein content and total and differential cell counts were analyzed, and BAL fluid were stored for inflammatory cytokines (interleukin-1β (IL-1β), interleukin-6 (IL-6), keratinocyte-derived cytokine (KC), macrophage inflammatory protein-1α (MIP-1α)) dosage by luminex kit (Milliplex® Map Kit, Millipore S.p.A., Vimodrone, Italy), according to manufacturer’s instructions.

Histology: histopathologic evaluations were performed in a blinded fashion by a pathologist (VC). Briefly, the lungs were excised, were fixed in 4% formaldehyde for 24 h (at a pressure of 20 cmH2O for the first 30 min), and then paraffin embedded and sectioned. Transverse sections (5 μm) were obtained by cutting the lungs from apex to base and then stained with hematoxylin and eosin (Sigma-Aldrich, St. Louis, MO, USA). Histopathologic examination was performed according to our previous study [25], evaluating the following six pathologic findings: alveolar serofibrinous exudate with hyaline membranes formation, alveolar hemorrhage, alveolar inflammatory cells, alveolar septa thickening, parenchymal necrosis, and fibrosis. Severity and extension of each pathologic finding were scored for severity as 0 (absent), 1 (mild), 2 (moderate), and 3 (marked) and for extension as 0 (absent), 1 (>0% and ≤25%), 2 (>25% and ≤50%), and 3 (>50%). A mean score for each finding for each lung was derived and expressed as the product of extent and severity.

### Late ARDS study: unilateral acid aspiration model

Rats were anesthetized with Ketamine 100 mg/kg and Xilazine 4 mg/kg, orotracheally intubated and ventilated (Inspira ASV, Harvard Apparatus, Holliston, MA, USA) with the same parameters used above. 0.1 M HCl (2.5 ml/kg) was instilled into the right bronchus through a PE10 catheter. The mechanical ventilation continued for 15 min and rats were maintained in the reverse-Trendelenburg position. Animals were extubated and kept in an oxygenated (FiO_2_ 0.5) chamber until full awakening. Rats were housed three/cage for 2 weeks in Specific Pathogen Free conditions, until sacrifice. A group of healthy rats (*n* = 6) did not undergo any of the surgical interventions and was sacrificed (Healthy rats).

### Treatment protocol

Administration of Ang-(1-7) or vehicle (NaCl 0.9%) started 10 min after the HCl instillation through the use of Alzet mini-osmotic pumps (Model 2002, DURECT Corporation, Cupertino, CA, USA), which were implanted and tunneled subcutaneously, with a mean pumping rate of 0.44 μl/h for 2 weeks until the rats were sacrificed. Throughout the study period, dosages were maintained as specified above. In this part of the study, we studied only on the dose (300 μg/kg/day) of Ang-(1-7), that demonstrated beneficial effects in the acute phase (see the ‘Results’ section). Ten rats were excluded from analysis (*n* = 7 vehicle, n = 3 Ang1-7) because of an evident inflammatory reaction localized near the subcutaneous mini-osmotic pump, which casts some doubts on the actual drug delivery and represents an additional source of inflammation.

### Assessment of injury

Blood oxygen saturation (SpO_2_): 1 week after the HCl instillation, the arterial hemoglobin saturation was measured through an animal oximeter pod (red and infrared light passed through pulsating blood in vascular tissue) placed on the hind leg (Animal Oximeter Pod, ML325, ADInstruments, Colorado Springs, CO, USA).

Respiratory system compliance: After 2 weeks of treatment in anesthetized rats, respiratory system compliance was measured through the construction of a PV curve: after a recruitment maneuver (to 30 cmH_2_O for 10 s), five steps of inspiratory volumes (2.5 ml) were delivered, starting from functional residual capacity to a total volume of 12.5 ml. For each step, the plateau pressure was recorded in order to calculate the static compliance.

CT scan: rats underwent two micro-CT scans (eXplore Locus, GE Healthcare, Pewaukee, WI, USA): 24 h after injury and at the end of the experiment. The CT parameters were as follows: voltage 80 kV, current 450 μA, and resolution 93 μm. The scans were acquired over 10 min. Images were analyzed to measure the amount of hypoaerated areas of parenchyma in the left and right lung separately.

Collagen content in pulmonary tissue: after animal’s euthanasia, lungs were excised and stored at −80°C. The collagen content was measured indirectly with the OH-proline assay. We used the conventional method, which entails lung tissue homogenization and hydrolysis with 6 N HCl at 120°C, chloramine T and Ehrlich’s solution were added to samples for the OH-proline oxidation and a colorimetric reaction. Absorbance was measured at 550 nm.

### Exclusion criteria

Animals were excluded from the protocol on fulfillment of two predefined criteria, namely: (1) evidence on CT of lung injury to the left lung following acid instillation (i.e., model failure); or (2) malfunctioning of the injection pumps (i.e., non-delivery of Ang(1-7)). In the case of animal death, this precluded further physiologic data collection, but animal survival data was included in the analysis.

### Statistical analysis

All data presented in this report are expressed as mean ± standard deviation. For the acute ARDS, study treatments with Ang-(1-7) at two doses and vehicle were compared by means of one way ANOVA analysis and followed by Tukey *post hoc* test. For the comparison between vehicle group and CTRL group, and between the two rescue treatment groups, *t*-test analysis was used. For the study of the late phase, angiotensin 1-7 was compared with vehicle by *t*-test analysis. A level of *p* < 0.05 was considered as statistically significant.

## Results

### Acute ARDS study. Two-hit model

#### Series 1 - early therapy with Ang-(1-7)

##### Angiotensin-(1-7) effects on pulmonary pathophysiology

We did not find any difference between groups in terms of hemodynamics and airway pressures during the experiment, as shown in Table 1.

**Table 1:** **Hemodynamics and airway pressure**

**Experimental groups (** ***n*** **)**	**Part (mmHg)**	**CVP (mmHg)**	**Paw (cmH**_**2**_ **O)**	**Ppeak (cmH**_**2**_ **O)**	**PEEP (cmH**_**2**_ **O)**
Vehicle (15)	112 ± 20	3.2 ± 0.9	5.7 ± 0.5	19.7 ± 2.0	2.3 ± 0.2
ALD (11)	117 ± 16	3.0 ± 0.8	6.0 ± 0.7	21.5 ± 3.5	2.2 ± 0.2
AHD (13)	123 ± 16	3.4 ± 0.8	5.8 ± 0.4	19.7 ± 1.5	2.4 ± 0.2

Part, arterial pressure; CVP, central venous pressure; Paw, mean airway pressure; Ppeak, peak of airway pressure; PEEP, positive end-expiratory pressure. Results are expressed as mean ± SD.

**Figure 1:**
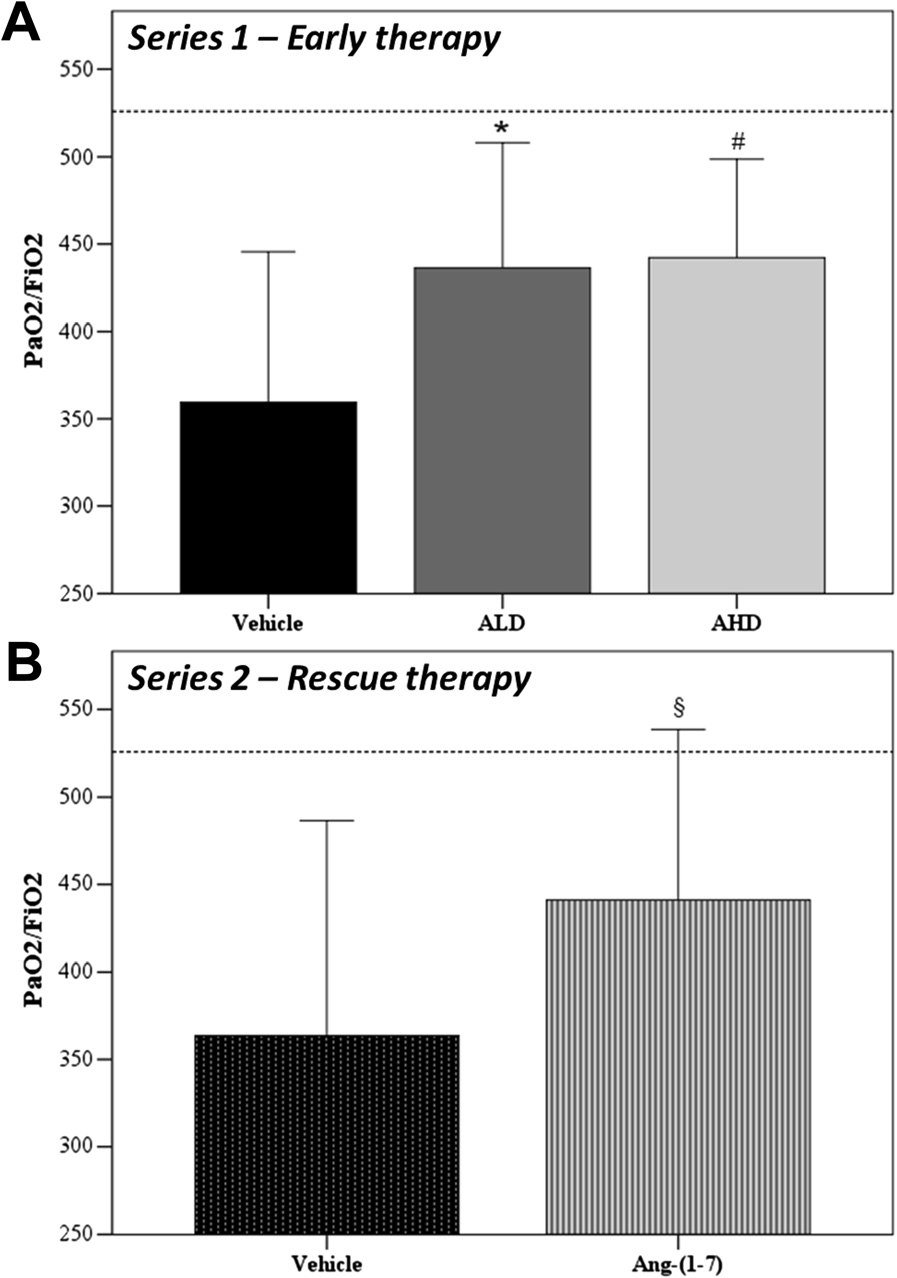
**Partial pressure of oxygen and fraction of inspired oxygen ratio (PaO2/FiO2) measured at the end of the protocol. (A)** Series 1 - Early therapy: ANOVA effect of treatment at the end *p* = 0.007; *p = 0.030 and #*p* = 0.013 vs vehicle. Vehicle *n* = 14; ALD *n* = 11; AHD *n* = 13. **(B)** Series 2 - Rescue therapy: §*p* = 0.043 vs vehicle. Vehicle *n* = 5; Ang-(1-7) *n* = 5. Healthy rats (dotted line). Results are expressed as mean ± SD.

Arterial oxygenation was similar in all groups immediately after acid instillation. At the end of the experiment, vehicle-treated rats showed a significant reduction in oxygenation compared to CTRL rats (554 ± 9 mmHg, *p* = 0.001). Ang-(1-7) treatment at both doses significantly attenuated the decrement in arterial oxygenation induced by acid instillation (Figure 1A) compared to vehicle treatment. Different treatments did not result in differences in terms of levels of carbon dioxide, pH, and BE.

**Figure 2:**
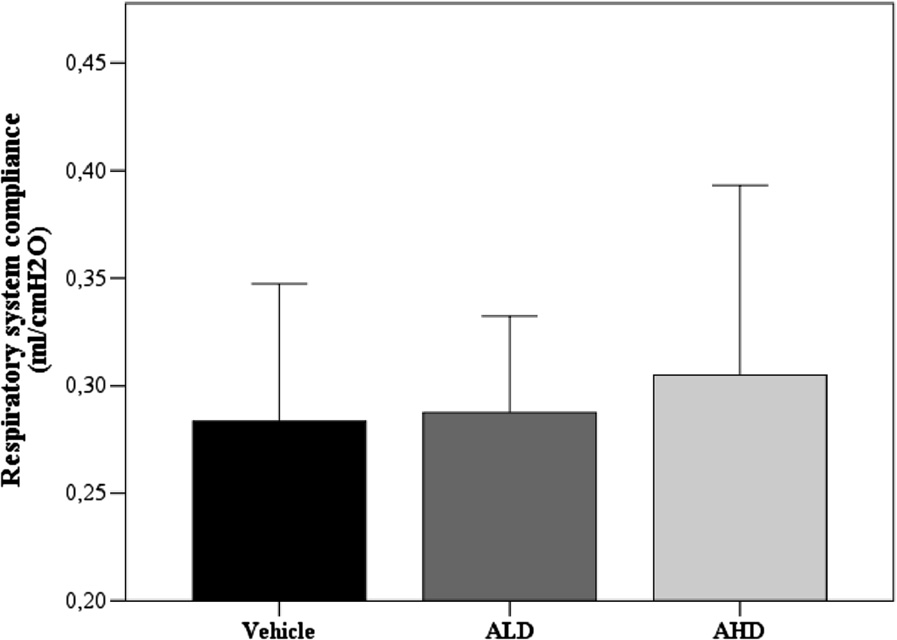
**Final respiratory system compliance in vehicle and Ang-(1-7) (ANOVA** ***p*** **= n.s.) treated groups.** Vehicle *n* = 20; ALD *n* = 12; AHD *n* = 14. Healthy rats = 0.65 ml/cmH_2_O. Results are expressed as mean ± SD.

As expected, lung compliance in vehicle group was significantly reduced if compared to CTRL rats (0.44 ± 0.03; *p* = 0.014). All groups immediately after acid instillation showed similar respiratory system compliance, and Ang-(1-7) treatment seemed not to affect the lung mechanical properties: all groups showed similar compliance values (Figure 2) measured at the end of experiment (5 h).

Rats that received saline instillation instead of acid showed a significant lower alteration (*p* = 0.019) of permeability than vehicle group in the right lung: total protein content was 722 ± 42 μg/ml in right and 692 ± 29 μg/ml in left lung. Ang-(1-7) treatment did not modify the alteration of alveolar permeability, as the total protein content was very similar in all groups, as shown in Table 2.

**Table 2:** **BAL total protein concentration and histological analysis in vehicle and Ang-(1-7) treated groups**

**Alveolar permeability and histology**	**Lung**	**Vehicle**	**ALD**	**AHD**
Permeability: BAL total protein content				
Protein content (μg/ml)	Right	2,454 ± 1,142	2,600 ± 844	2,536 ± 710
	Left	1,108 ± 668	1,406 ± 808	1,213 ± 610
Histological analysis				
Alveolar serofibrinous exudate	Right	1.4 ± 0.5	2.0 ± 2.7	1.2 ± 0.9
	Left	1.0 ± 1.0	1.0 ± 1.0	0.3 ± 0.6
Alveolar hemorrhages	Right	0.7 ± 0.9	0.7 ± 1.1	0.7 ± 1.7
	Left	0 ± 0	0 ± 0	0 ± 0
Alveolar inflammatory cells	Right	0.8 ± 0.8	0.4 ± 1.2	0.5 ± 0.8
	Left	0 ± 0	0 ± 0	0 ± 0
Parenchymal necrosis	Right	1.6 ± 3.1	1.2 ± 2.3	1.2 ± 2.6
	Left	0.3 ± 0.6	0 ± 0	0 ± 0

Results are expressed as mean ± SD.

##### Angiotensin-(1-7) effects on inflammation

**Figure 3:**
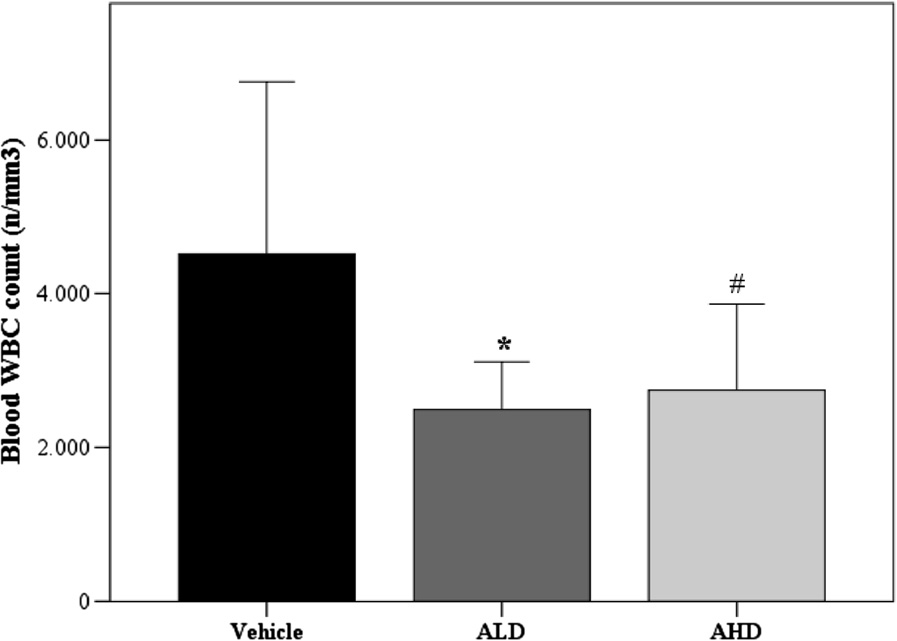
**Peripheral white blood cells counts at the end of experiment (after 5 h).** ANOVA, effect of treatment *p* = 0.004. *p = 0.006 and #*p* = 0.025 vs vehicle. Vehicle *n* = 15; ALD *n* = 12; AHD *n* = 10. Results are expressed as mean ± SD.

**Figure 4:**
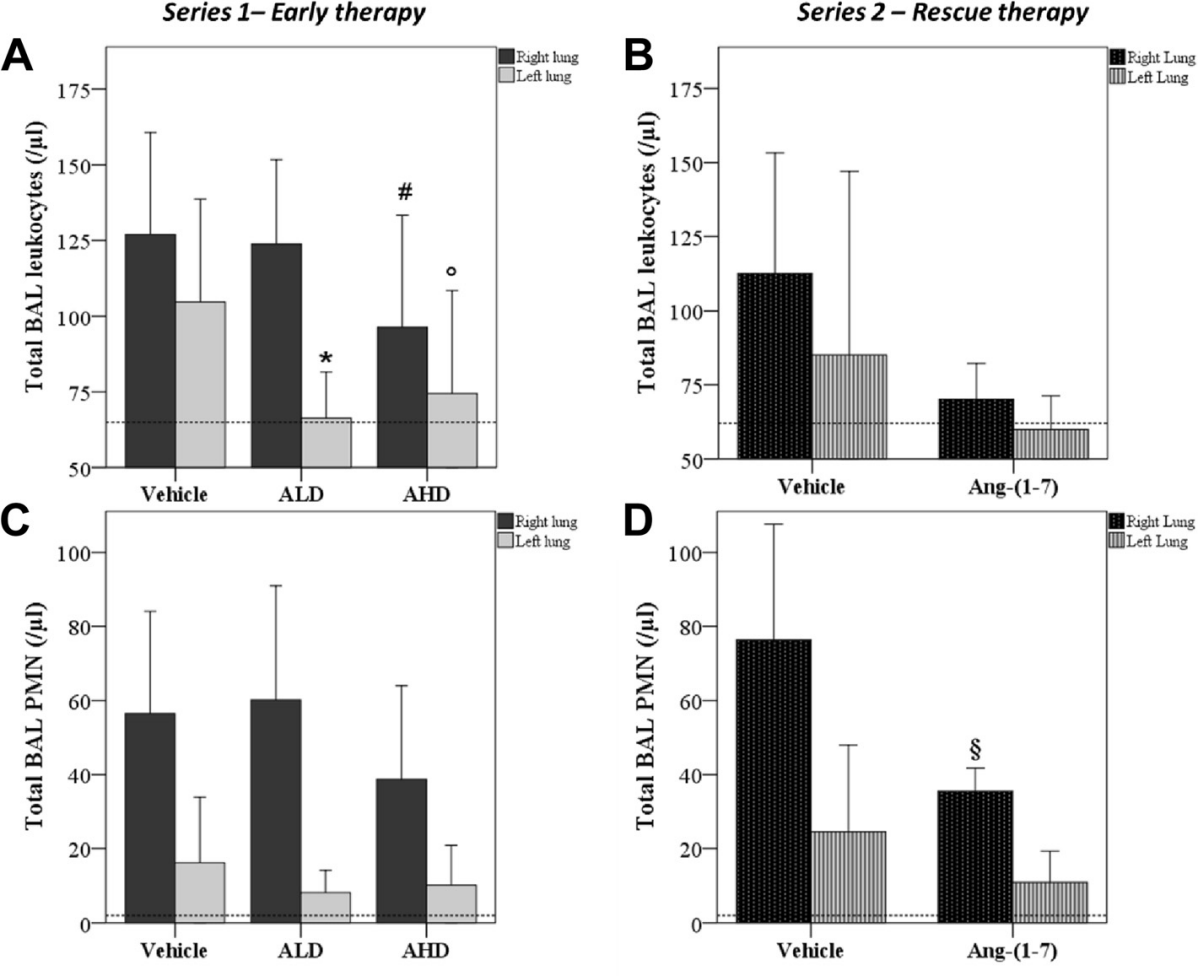
**Total cells recruited in alveolar spaces. (A)** Series 1 - Early therapy: in right (ANOVA *p* = 0.035) and left (ANOVA *p* = 0.004) lung. *p = 0.033 vs vehicle, #*p* = 0.006 vs vehicle and °*p* = 0.034 vs vehicle in the same lung. **(B)** Series 2 - Rescue therapy. Vehicle *n* = 5; Ang-(1-7) *n* = 5. Total PMN recruited in alveoli. **(C)** Series 1 - Early therapy: in right (ANOVA *p* = n.s) and left (ANOVA *p* = n.s). Vehicle *n* = 16; ALD *n* = 11; AHD *n* = 11. **(D)** Series 2 - Rescue therapy: §*p* = 0.021 vs vehicle. Vehicle *n* = 5; Ang-(1-7) *n* = 5. Healthy rats (dotted line). Results are expressed as mean ± SD.

As index of the systemic inflammation, peripheral white blood cell counts were performed: cellular counts were similar in all groups immediately after injury (vehicle: 8,271 ± 3,054; ALD: 8,681 ± 2,919; AHD: 9,085 ± 2,479/mm^3^) and after 3 h (vehicle: 4,536 ± 2,211; ALD: 3,071 ± 1,453; AHD: 3,785 ± 1,622/mm^3^), but after 5 h of treatment with Ang-(1-7) at both doses, the number was significantly reduced, compared to vehicle treatment (Figure 3). As a measure of the alveolar inflammatory response, we evaluated the alveolar cell recruitment and the cytokine levels. Ang-(1-7) treatment reduced the number of inflammatory cells recruited in BAL. In particular, the high dose treatment led to a lesser number of total cells in BAL in both lungs, whereas the low dose affected only the left lung (not directly injured by acid) (Figure 4A). Rats in CTRL group had significantly lower (*p* = 0.045) polymorphonuclear (PMN) count in BAL in the right lung (right lung: 15 ± 5/μl, left lung: 6 ± 3/μl) when compared to the vehicle treatment. While the number of PMN in BAL seemed to be not reduced in AHD group compared to the vehicle (Figure 4C).

We did not find any effect of Ang-(1-7) on cytokine BAL levels, as shown in Table 3.

**Table 3:** **BAL cytokines dosage (pg/ml) in vehicle and Ang-(1-7) treated groups **

**Experimental groups (** ***n*** **)**	**IL-1β (pg/ml)**		**IL-6 (pg/ml)**		**KC (pg/ml)**		**MIP-1α (pg/ml)**	
	**Right**	**Left**	**Right**	**Left**	**Right**	**Left**	**Right**	**Left**
Vehicle (14)	68 ± 32	40 ± 29	3,423 ± 1,460	1,697 ± 2,112	2,133 ± 608	1,337 ± 895	45 ± 35	20 ± 15
ALD (10)	91 ± 47	68 ± 57	3,771 ± 1,647	1,958 ± 2,964	3,003 ± 1,257	1,900 ± 1,472	67 ± 39	22 ± 16
AHD (9)	83 ± 23	38 ± 28	4,465 ± 1,972	1,300 ± 554	2,809 ± 1,198	2,366 ± 999	54 ± 22	22 ± 16
Healthy rats (6)	27 ± 10	15 ± 6	253 ± 220	357 ± 78	233 ± 106	104 ± 43	15 ± 10	10 ± 8

Results are expressed as mean ± SD. (ANOVA *p* = n.s.).

##### Histology and imaging

**Figure 5:**
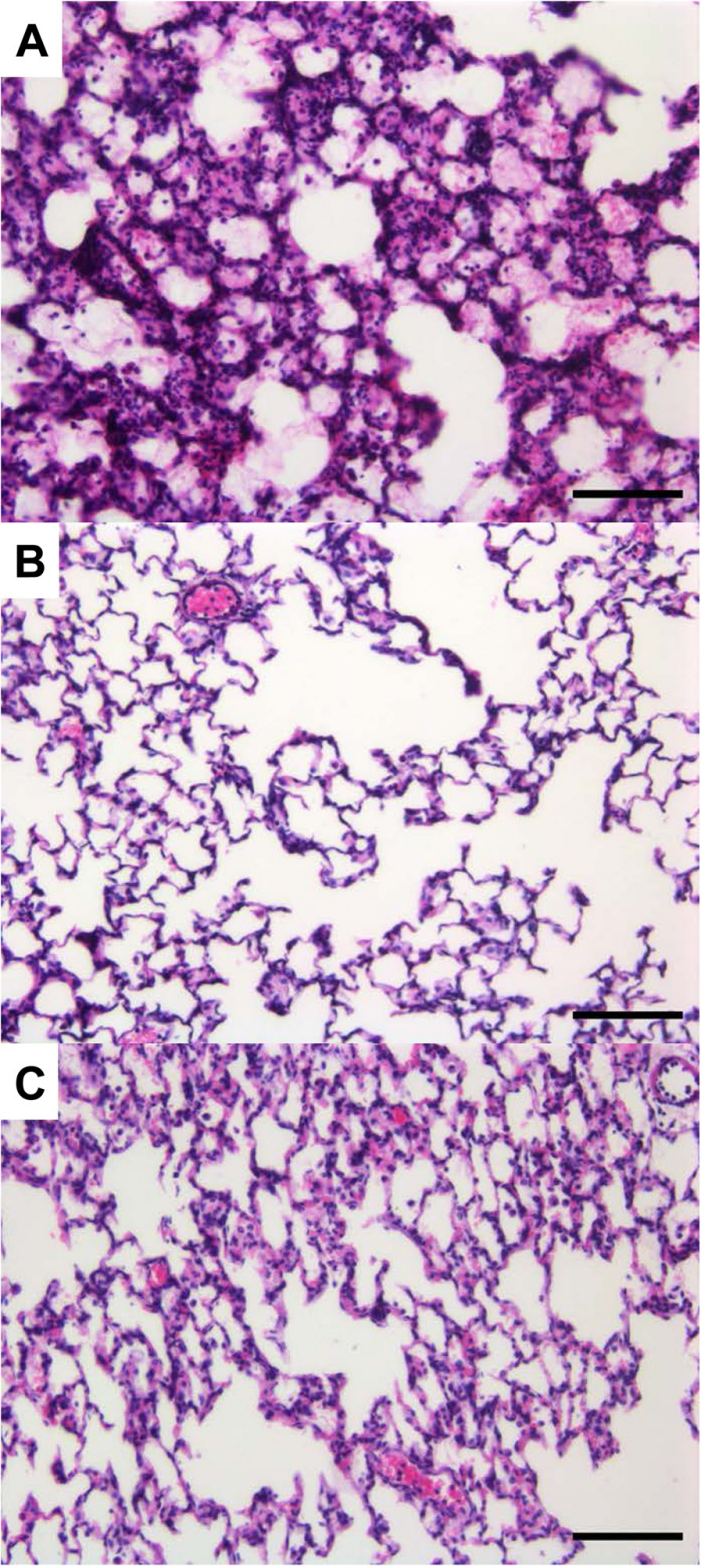
**Major histopathological findings. (A)** Vehicle. Within the lumina of alveoli, there are mats of pale eosinophilic finely beaded to fibrillar material (fibrin), in which are embedded moderate numbers of both viable and degenerated (karyorrhectic) neutrophils. **(B)** ALD. No inflammatory cells detectable within alveolar lumina. **(C)** AHD. Note within the lumina of slightly compressed alveoli scattered neutrophils (right lung, hematoxylin-eosin, 200×, scale bar = 100 μm).

Major histopathological findings are summarized in Table 2 and Figure 5. A trend towards reduced alveolar inflammatory infiltrates, parenchymal necrosis and serofibrinous exudate, although not reaching statistical significance was noted in Ang-(1-7) treated group. The extent of alveolar hemorrhage was similar in all treatment groups.

**Figure 6:**
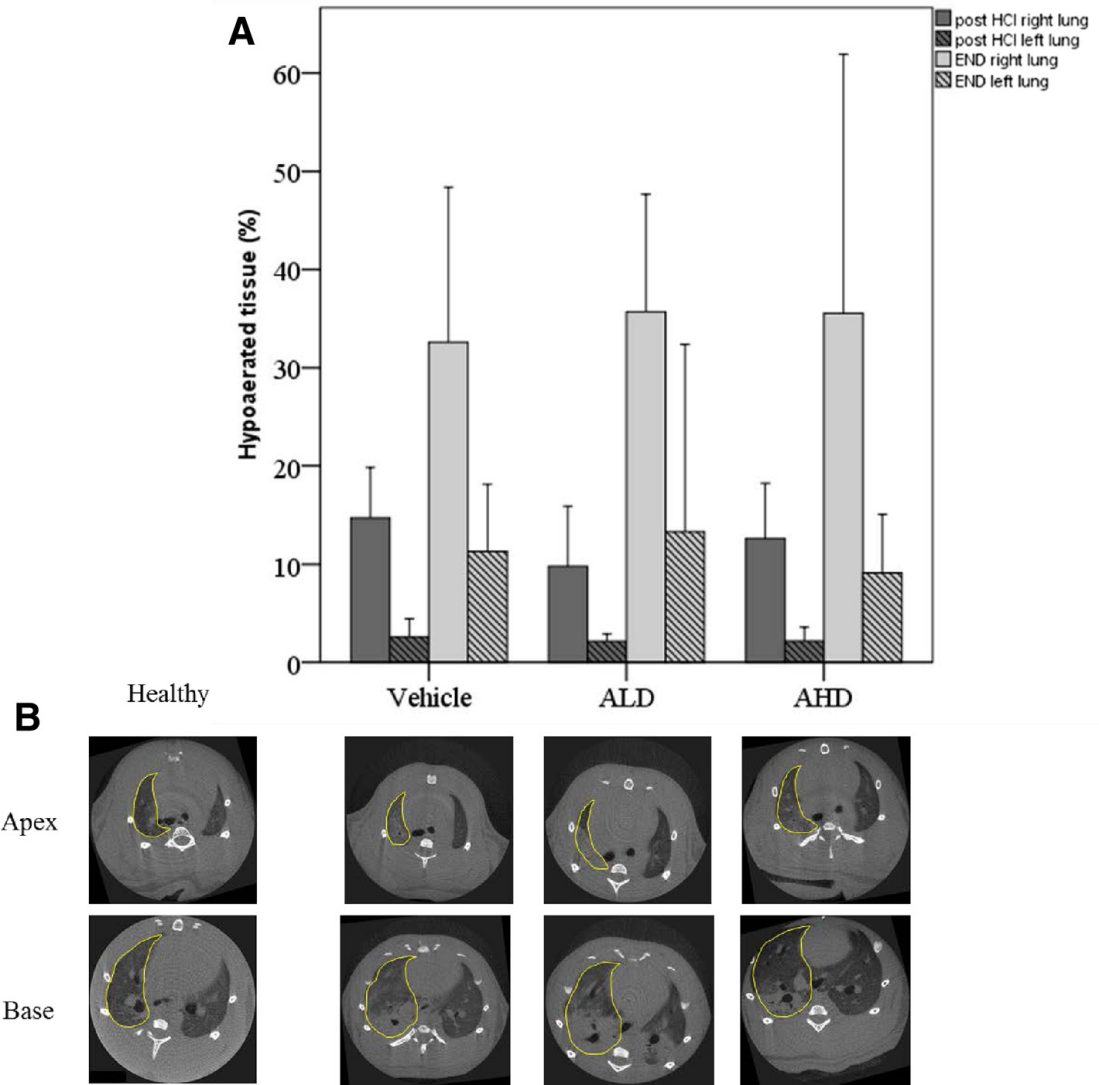
**Percentage of hypoaerated areas and representative CT images. (A)** Percentage of hypoaerated areas of parenchyma measured on CT image obtained immediately after HCl instillation and at the end of experiment. Vehicle *n* = 9; ALD *n* = 7; AHD *n* = 6. **(B)** Representative CT images at the apical and basal level in Healthy rats, vehicle, ALD and AHD groups. Regions of interest for hypoaeration measurement in right lung are outlined.

No differences were found in terms of percentage of hypoaerated areas between groups, neither immediately after HCl instillation nor at the end of experiment (Figure 6).

#### Series 2 - rescue therapy with Ang-(1-7)

In series 2, Ang-(1-7) was effective in improving oxygenation and also if administrated as rescue treatment 2 h after the injury (*p* = 0.043), as shown in Figure 1B. Moreover, the rescue treatment with Ang-(1-7) induced a significant decrease (*p* = 0.021) in alveolar PMN in the right lung if compared to vehicle treatment, while no difference was detected in the left lung (Figure 4D). We also observed the effect of Ang-(1-7) on diuresis: the urine volume increased significantly if compared to vehicle group (vehicle: 2.9 ± 0.6 ml/5 h, Ang-(1-7): 7.9 ± 4.2 ml/5 h; *p* = 0.030)), which was not significant in series 1 (data not shown).

### Late ARDS study. Unilateral acid aspiration model

#### Angiotensin-(1-7) effects on lung fibrotic evolution

**Figure 7:**
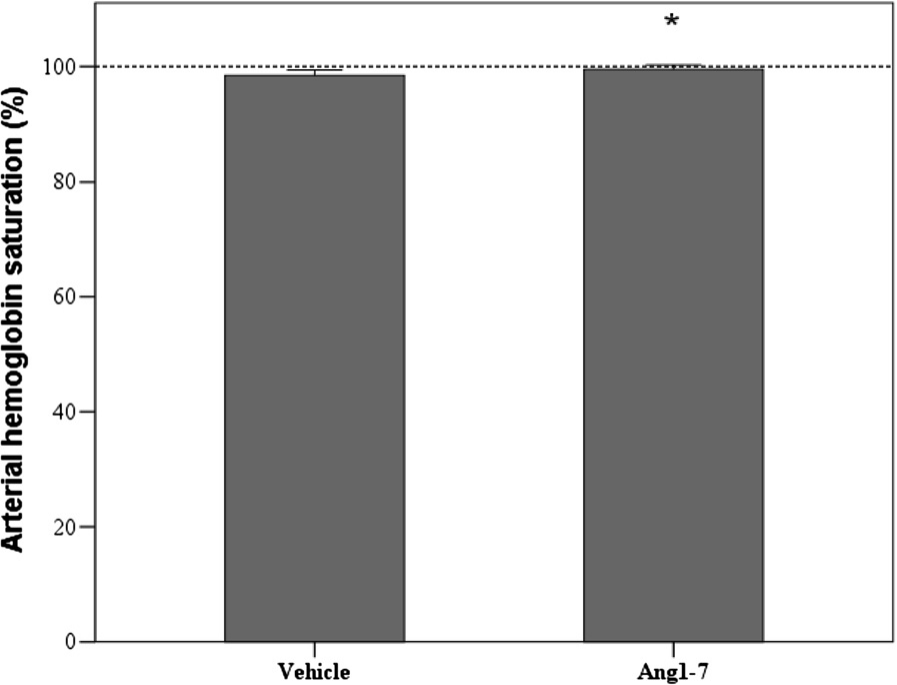
**Arterial hemoglobin saturation in vehicle an Ang1-7 group.** *p = 0.032 vs Ang1-7. Vehicle *n* = 7; Ang-(1-7) *n* = 8. Healthy rats (dotted line). Results are expressed as mean ± SD.

One week after acid instillation, blood oxygen saturation was significantly higher in Ang-(1-7) group compared to that in the vehicle group, as shown in Figure 7. Albeit the difference was minimal, it reinforces the finding of an improved oxygenation reported in the acute phase.

The lung mechanical properties measured at the end of experiment were not different between two groups: vehicle 0.65 ± 0.14 and Ang-(1-7) 0.62 ± 0.06 ml/cmH_2_O. Healthy rats showed a respiratory system compliance of 0.73 ± 0.04 ml/cmH_2_O.

**Figure 8:**
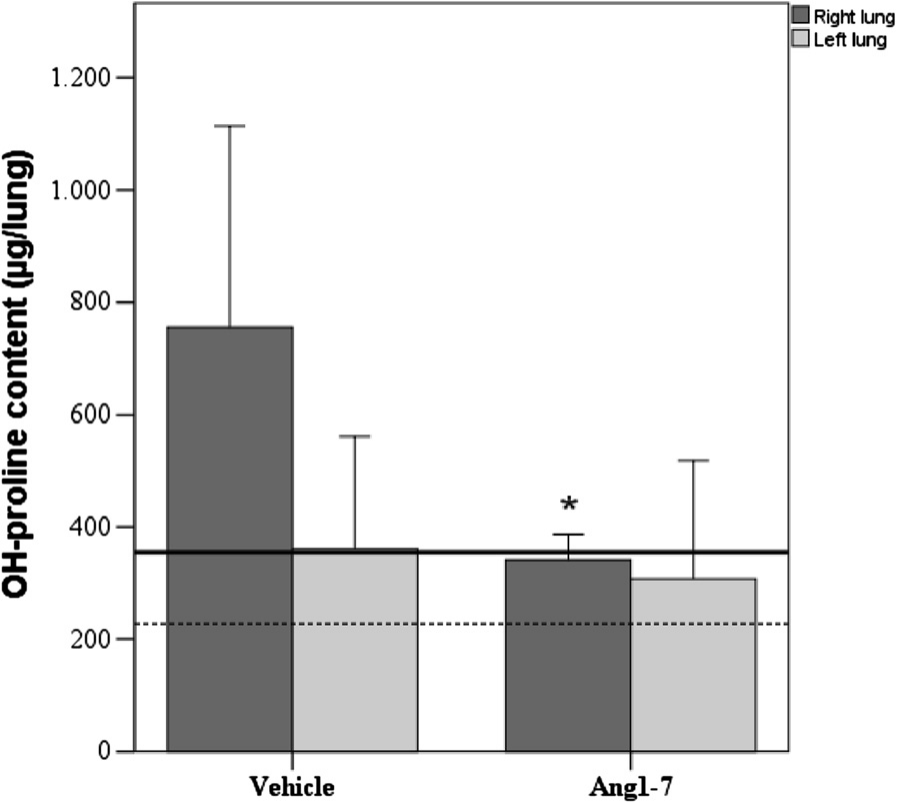
**OH-Proline content in vehicle and Ang1-7 group.** OH-Proline content in right and left lung shown separately, *p = 0.006 vs Ang1-7 right lung. Vehicle *n* = 7; Ang-(1-7) *n* = 8. Healthy rats: right lung (solid black line) and left lung (dotted black line). Results are expressed as mean ± SD.

At the end of experiment, right lungs from rats treated with angiotensin-(1-7) showed a significant reduction in collagen deposition, indirectly measured through OH-proline assay (Figure 8).

## Discussion

The RAS plays an important role in the pathogenesis of ARDS; in particular, it can be considered as an important element in the inflammatory response, since angiotensin II acts as a growth factor regulating cell growth and fibrosis [3]. The ACE2/Ang-(1-7) axis exerts protective effects in several pathological scenarios in different organs, by opposing the overactivation of ACE/Ang-II axis [19]. The aim of this study was to test the efficacy of angiotensin-(1-7) administration as treatment of ARDS in the two phases of the disease, the acute and the late in an experimental model of acid-induced lung injury.

To this end, we used our recently published two-hit model of acute lung injury [27] induced by unilateral acid instillation and prolonged injurious ventilation in these studies. A key feature of this model is the presence of a lung (the right one) subjected to a double insult (acid aspiration and injurious mechanical ventilation) and the other lung (left) that only receives injurious mechanical ventilation. This model is a valid model of acute lung injury since it fulfilled the criteria laid out by Matute-Bello et al. [28]: histological evidence of tissue injury, alteration in the alveolar capillary barrier, presence of inflammatory response, and evidence of physiological dysfunction. It is known that high tidal volume ventilation causes VILI after acid instillation in rats [29]: however, to show that this model was more injurious than prolonged mechanical ventilation alone, rats treated with sterile saline instead of hydrochloric acid and mechanically ventilated for 5 h were used as control. Our results demonstrated that the two-hit model showed a significant alteration of pulmonary function (gas exchange and respiratory system compliance), increase in PMN alveolar recruitment as index of inflammatory response, and alteration of alveolar capillary barrier. This model resembles to some extent the inhomogeneity of ARDS in the clinical setting. Indeed in the same subject, it’s possible to study an area injured by acid instillation and mechanical ventilation (right lung) and at the same time a region injured by mechanical ventilation only (left lung).

Ang-(1-7) administration proved to be safe and well tolerated in our experimental setting: only a slight and transient (10 to 15 min) decrease in systemic blood pressure was detected in the acute phase study, confirming the vasodilator effect of Ang-(1-7) which has been shown to be mediated through the production of endothelial nitric oxide [30]. We found no difference in survival in both ARDS studies: almost 80% of animals in all groups survived until the end of experiments.

The main findings obtained in the study are the beneficial effects of Ang-(1-7) on the oxygenation, inflammatory cells infiltrates, and fibrosis. The Ang-(1-7) treatment attenuated the decrease in gas exchange in rats subjected to acid instillation and 5 h of injurious mechanical ventilation: rats treated with Ang-(1-7) showed better partial pressure of oxygen compared to rats treated with vehicle only (an increase of 22%). Interestingly, this finding was confirmed in the late ARDS model, 1 week after the acid instillation, as demonstrated by the measurement of blood oxygen saturation. It should be stressed that while this improvement (99.4% vs 98.5%) is certainly not clinically relevant, mostly because of the limited degree of hypoxia in this long-term model, at the same time, it supports the positive data obtained in the acute phase study. However, the lung function improvement did not seem to reflect on pulmonary mechanical properties. In fact, no improvement in respiratory system static compliance was detected in Ang-(1-7) higher dose group in acute phase study.

Inflammatory cell migration was significantly reduced in Ang-(1-7) groups: both numbers of white blood cells and total BAL cells (Figures 3 and 4A) are significantly lower than in the vehicle group, although only a slight reduction was evident in PMN count (Figure 4B). A reduced systemic spread of the local inflammatory stimulus could also explain the decreased peripheral white blood cells; at the same time, Ang(1-7) is known to have direct effects on erythropoiesis [31], although this mechanism is less likely due to the limited time of observation. Moreover, histological analysis showed a reduced although not statistically significant presence of alveolar inflammatory cells (Table 2). Overall, these results confirm Ang-(1-7)-mediated inhibition of leukocyte migration in different pathologies, such as acute renal injury [32], antigen-induced arthritis [17], and cardiomyopathy [21]. Moreover, a very recent study performed by Klein and coworkers [24] demonstrated how Ang-(1-7) protects from three experimental lung injury: oleic acid, acid aspiration, and mechanical ventilation. It clearly attenuated alveolo-capillary barrier failure, neutrophil influx into the lung, and histological evidence of lung injury. In contrast to our results on inflammatory cell lung influx, we failed to find any difference in BAL levels of different cytokines (IL-1β, IL-6, KC, and MIP-1α). In fact, it seemed that Ang-(1-7) treatment did not affect cytokine production. These results are in line with those obtained by Arndt et al. in 2006 [33], that showed that the diminution in neutrophil recruitment in lung was not dependent on a decrease in neutrophil chemoattractants (i.e,. KC and MIP-2) in the setting of systemic ACE inhibition in lypopolysaccharide-induced lung injury in mice. A possible hypothesis that can be drawn to explain this finding is the involvement of nitric oxide (NO). Ang-(1-7) can induce the activation of endothelial NO synthase (eNOS) [34] and the bradykinin-mediated NO-release [35]. NO, besides its vasodilatory and anti-aggregant functions, inhibits neutrophil recruitment, by acting on neutrophil movement in vascular bed and on the neutrophil-endothelial interactions, via the suppression of adhesion molecules [36]. Although Klein et al. [24] found a significant protective effect of Ang-(1-7) on alveolo-capillary barrier, the total BAL protein content in our experimental setting was not different between the analyzed groups. One of the reasons for the lack of the effect on protein extravasation, in our study, could be related to the experimental model: the caustic action of acid direct on a limited area (right lung) and the following prolonged mechanical ventilation may cause such an intense fluid extravasation into the alveolar space that the 5-h time period could not be sufficient for the treatment to exert its entire effect.

The therapeutic potential of Ang-(1-7) treatment was underlined in a separate set of experiments (series 2), where ‘rescue therapy’ starting 2 h after HCl instillation demonstrated a significant improvement of arterial oxygenation and inflammatory response (in terms of PMN recruitment into alveoli). This is probably one of the major elements of novelty of this paper, when compared to the paper by Klein [24], in adjunct to the evaluation on the long-term development of fibrosis and the use of a two-hit model. Interestingly in this experiment, we found a role of Ang-(1-7) on diuresis: it seemed that a higher infusion rate of Ang-(1-7) (100 μg/kg/h) induced an increase in urine volume if compared to vehicle treatment, confirming data in literature [37].

Another very interesting result is the beneficial effect of Ang-(1-7) treatment on the fibrotic evolution. In the second part of the study (late ARDS study), we tested the effects of the Ang-(1-7) only at the dose that had produced the better effects in the acute ARDS study. Two weeks after acid instillation, rats continuously treated with Ang-(1-7) had a significant reduction in collagen lung deposition (indirectly measured by OH-proline assay), confirming recent studies performed by Chen et al. [38] and Meng et al. [39], that demonstrated protective effects of Ang-(1-7) treatment in murine LPS- or bleomycin-induced lung injury and lung fibrosis. The inhibitor effect of Ang-(1-7) on inflammatory cells recruitment seen in the acute phase study may be related to the reduction of fibrosis in the later phase. We have recently published [40] about the relationship between the inflammatory response and the fibrotic evolution, confirming previous data by Jones et al. [41]. Although Ang-(1-7) decreased pulmonary collagen deposition, it did not attenuate the decrement in respiratory system compliance in these animals. This might be explained with the fact that lung injury is restricted to a single lobe, and it is not so big to affect the whole respiratory system compliance.

The limitations of this study should also be acknowledged. First, we did not include a group of animals treated with blockers of Ang-II receptor (such as Losartan) or, vice-versa of Ang-II. Indeed, several studies showed beneficial effects of Losartan treatment in terms of attenuation of lung injury in the ARDS model [33,38,42-44]. Second, we did not demonstrate that the effects of Ang-(1-7) are mediated by Mas receptor, since we did not consider a group of animals treated with Mas receptor antagonist (A779). Ang-(1-7) mediates its lung effects through its own receptor and A779 exacerbates lung injury and collagen deposition [24,40,41].

## Conclusions

In this study, angiotensin-(1-7) improved pulmonary function in terms of oxygenation and inflammatory cells recruitment in a two-hit model of ARDS, characterized by acid instillation and prolonged injurious ventilation. Ang-(1-7) was effective even when administration was delayed, emphasizing its true therapeutic potential. The more interesting finding, from a potential clinical perspective, is the persistence of an improved oxygenation and a reduced lung fibrosis, evaluated in the experimental model of acid aspiration alone. These results make Ang-(1-7) an exciting plausible therapy for ARDS and call for further research in this field.
